# Default Mode Network Efficiency Is Correlated With Deficits in Inhibition in Adolescents With Inhalant Use Disorder

**DOI:** 10.3389/fpsyt.2020.00209

**Published:** 2020-03-26

**Authors:** Dailett M. Hernández-Álvarez, Lucero Pacheco, Roberto Velasco-Segura, Miguel Pérez de la Mora, Claudia Tejeda-Romero, Nadia González-García

**Affiliations:** ^1^Department of Cognitive Neuroscience, Instituto de Fisiología Celular, Universidad Nacional Autónoma de México, Mexico City, Mexico; ^2^Instituto de Ciencias Aplicadas y Tecnología, Universidad Nacional Autónoma de México, Mexico City, Mexico; ^3^Centros de Integración Juvenil, A.C., Mexico City, Mexico; ^4^Laboratory of Neurosciences, Hospital Infantil de México Federico Gómez, Mexico City, Mexico

**Keywords:** executive-function, resting-state functional magnetic resonance imaging, substance use disorder, functional connectivity, adolescents

## Abstract

It is well established that alterations in cognitive function and damage to brain structures are often found in adolescents who have substance use disorder (SUD). However, deficits in executive cognitive functioning in adolescents related to the vulnerability and consumption of such substances are not well known. In this study, we use graph theoretic analysis to compare the network efficiency in the resting state for three networks—default mode network (DMN), salience network (SN) and fronto-parietal network (FPN)—between inhalant-consuming adolescents and a control group (12 to 17 years old). We analyzed whether the efficiency of these functional networks was related to working memory, mental flexibility, inhibition of response, and sequential planning. We found that, when compared to the control group, inhalant-consuming adolescents presented with important deficits in communication among brain regions that comprise the DMN, SN, and FPN networks. DMN is the most affected network by inhalant abuse during adolescence. The mediation analyses suggested that the relationship between inhalant abuse and inhibitory control and sequential planning was partly mediated by DMN efficiency.

## Introduction

The consumption of illicit drugs by young people has increased in recent years, becoming a public health issue in several countries (1). Numerous clinical and epidemiologic studies [*e.g.*, (2–4)] have indicated that cognitive and emotional effects are more severe and persistent if their use begins during childhood or adolescence rather than if it happens in adulthood. Thus, research on addiction in the context of a developing brain is currently of utmost importance. Prevention and early intervention programs are needed to face the growing indexes of addictive behavior among the young population.

A recent study showed that many individuals with addiction present neuropsychological deficits across a range of functions: inhibition, compulsivity, action selection, expectancy, and reward learning and valuation. These functions could be linked with vulnerability to and the treatment and diagnosis of substance use disorders (SUDs) (5).

Several clinical investigations in adults, including those in subjects with chronic job-related industrial solvent exposure, have shown that chronic use of inhalants is associated with significant brain abnormalities. Among the most commonly reported effects are leukoencephalopathy (6, 7) and atrophy of both the cerebellum and corpus callosum (8). It is worth mentioning that inhalants are commonly one of the earliest drugs used by adolescents, and a few authors have pointed out that such encephalopathies can be observed in young consumers (9, 10).

Neuropsychological research has contributed to relating the brain damage associated with inhalant consumption with alterations in the cognitive function of adolescents. Thus, it is interesting to note that Takagi and collaborators [see (11, 12)] have reported that inhalant users show, when compared with consumers of other drugs and controls, the worst performance in tasks that measure attention, memory, cognitive control, and processing speed. In line with this, (13, 14) have also described more significant deficits in working memory, processing speed, concept formation, and mental flexibility in adolescents that have used several drugs, including inhalants, compared to adolescents with no inhalants in their consumption history.

Complex cognitive processes, as previously mentioned, involve the integration of information through functional communication between anatomically distributed brain regions (15). Recently, a number of neurocognitive disorders have been studied using functional magnetic resonance imaging (fMRI) from the perspective of complex disorders of functional connectivity. Those disorders include autism (16, 17), attention-deficit disorder (18, 19), and dementia (20, 21). In addition, functional connectivity alterations have also been revealed in regard to some aspects of addictive behavior (22–25). We know that inhalants affect anatomical connectivity. It is yet unknown, however, what functional connectivity networks are affected, although we know that functional connectivity is constrained by anatomical structural connections (26). Structural connections are affected in inhalant users (10), particularly during adolescence, when a large-scale refinement of the functional networks takes place (27).

In this case–control study, we explored the performance of complex cognitive functions in inhalant-consuming adolescents and whether intranetwork communication efficiency is acting as a mediator of the effects of consumption on executive cognitive functions. We decided to examine executive functions to identify deficits with SUD as in previous studies. Based on the results of previous inhalant abuse studies, in which damage to structural connections and cognitive deficits were linked to inhalant use, we hypothesized that inhalant abuse during adolescence which is a maturational period of executive function (EF) and the brain, affects functional brain networks’ topologies that would lead to cognitive deficits. We focused on three functional networks that have received special attention in the investigation of the neural correlates of executive function development. Recently, studies have shown that modular segregation of the fronto-parietal network (FPN) and global efficiency mediated improvements in executive functions with age in structural networks and further that reciprocal activation of the FPN and deactivation of the default mode network (DMN) as well as the flexible transition between these networks are correlated to executive function across adolescence (28–30). Recent evidence suggests the switch between the DMN and FPN is modulated by the anterior insula which is an important region in the salience network (SN) (31). Graph theoretic analysis was applied to the analysis of functional connectivity. This method is based on the conception of the brain as a complex and efficient “small world” network, which can be qualitatively described through a variety of measurements (32–34).

## Methods

### Participants

A total of sixty subjects participated in this study: 30 inhalant-consuming adolescents (IC) and 30 healthy controls (HC), with ages ranging from 12 to 17 years. Three HC were excluded from the study after the fMRI due to image processing difficulties related to excessive movement of the subject during image acquisition. Age (IC: 15.1 ± 1.3 years, HC: 15.0 ± 1.4 years (= 0.71, Student’s t-test) and sex (IC: 24 men/6 women, HC: 16 men/11 women; p = 0.15, chi-square test) showed no significant differences between the two groups.

Inhalant use was characterized by structured interviews. The adolescent consumers had a diagnosis of mild or moderate SUD according to the Diagnostic and Statistical Manual for Mental Disorders (DSM)-V. All of the IC had been systematic inhalant users (more than three times a week) for at least a year. Most of them were polydrug consumers, with a preferential use of inhalants (see **Table 1**). Eighty percent of the consumers reported the use of solvents (paint thinners, acetone, and gasoline), and 20% also used aerosols and adhesives (paints, deodorants, and glues). The subjects were in the first week of remission under pharmacological treatment to minimize the effect of these compounds on cognitive performance and neurovascular coupling in the brain.

**Table 1:** Basic demographic and substance use histories for the inhalant and control groups.

		Inhalant group (IC) n = 30	Control group (HC) n = 27
Age	Mean (SD)	15.1 (1.3)	15.0 (1.4)
Years of education	Mean (SD)	8.1 (1.1)	9.9 (1.4)
Sex	% male	80	59
Age of first self-reported inhalant use (in years)	Mean (SD)	13.03 (1.6)	N/A
Duration of regular inhalant use (in months)	Mean (minimum–maximum)	24 (12–60)	N/A
Duration of regular use (months): Tobacco Alcohol Cannabis Cocaine	Mean (minimum–maximum)	20 (1–72) 18 (4–60) 16 (6–48) 4 (1–36)	N/A

None of the subjects had a history of premature birth, birth weight lower than 2.5 kg, or had suffered a neurologic disease, according to a neurodevelopment interview conducted with the parents. Additional exclusion criteria were the following: a) I.Q. lower than 70 (intellectual disability); b) lack of a family environment to avoid family abandonment and homeless situations; c) sensorial and motor disabilities; and d) any condition that prevented the use of a magnetic resonator, such as metallic implants, pregnancy or claustrophobia.

### Procedure

The adolescents comprising the IC were engaged in the study through the Centers for Youth Integration, while the HC came from high and senior high schools, which were all in Mexico City. All IC were recruited by professionals with experience on addictive disorders through a complete assessment based on the DSM-V, taking into account the study’s objectives and eligibility criteria. All subjects underwent a consent process in the presence of a family member, where they received detailed information regarding confidentiality, risks and benefits, psychometric tests, length of the study, *etc*. For the IC, relevant information regarding the use of addictive substances was also acquired in the same session by a structured interview. On this first encounter, either the Wechsler Intelligence Scale for Children (35) or the Wechsler Adult Intelligence Scale (36) was applied depending on the age of the subject. Family members were also interviewed in the same session to gain insight regarding their neurodevelopmental characteristics.

Both neuropsychological tests and resting-state fMRI acquisition were applied during a second session to all volunteers who fulfilled the eligibility criteria. All the procedures of the protocol were approved by both the Scientific Research Committee of the Centers for Youth Integration and the Ethics Committee of the Children’s Hospital of Mexico “Federico Gomez”.

### Cognitive Functioning Assessment

Four complex cognitive functions, known as executive functions due to their role in the generation, regulation, execution, and readjustment of behaviors, were studied to obtain information on the subject’s short-, medium- and long-term goals (37, 38), as these measures can be potentially affected in inhalant users (13, 39). We used the Neuropsychological Battery of Executive Functions and Frontal Lobes (BANFE), which consists of a number of worldwide-validated cognitive tests. The Mexican standardization sample was 500 children and adolescents who were divided by age into five groups; internal consistency reliability had a mean of.85 for test (40), and since age and education have been shown to have an important influence on executive performance, individual scores were transformed to standardized scores following norms for the Mexican population that take both characteristics into account.

- **Verbal working memory:** This function, which involves the ability to maintain and manipulate verbal information, was assessed by using the Working Memory Index from the Wechsler intelligence scales.
- **Mental flexibility:** This function was assessed with the Wisconsin Card Sorting Test (WCST), which allows an assessment of the mental flexibility of an individual through his/her capacity to change a classification criterion when it is inadequate. In the test, the subject must take one card from a deck—the card may contain figures with different colors, geometrical shapes and numbers—and to choose were to place it according to a changing classification criterion. For this study, the total number of correct answers was used (41).
- **Cognitive control:** The capacity to inhibit an automatic response was assessed by the application of the Stroop test. The test measures the inhibition abilities and the resistance to interference related to external stimuli through the capacity of the individual to inhibit the highly automated habit of reading every time a word is presented to him/her (42). Volunteers are presented with printed words, ordered in columns and are instructed to describe the color of the word and not read the word when they were underlined. The number of errors according to the given instructions was registered and used as the performance of the subject in this test.
- **Action planning and sequencing:** This cognitive function was assessed with the Tower of Hanoi test (TOH), which requires sequential planning of upcoming intermediate steps to achieve a final goal. The test consists of moving discs from an initial state to a finished state in the smallest number of steps as possible while following a specific set of rules: a) only one disc can be moved at the time; b) the discs can only be moved to a different plug and c) a disc cannot be placed on top of another disc that is smaller than itself (43). There are two levels of complexity, with three discs at the first level and four discs at the second level. The total number of movements in the task with the maximum level of complexity was considered the performance measure.

## Resting-State fMRI Data Acquisition and Preprocessing

The fMRI data were acquired while the subjects remained silent and stared at a cross mark on a Siemens Trio 3.0 Tesla MRI scanner at the National Institute of Neurology and Neurobiology “Manuel Velasco Suárez” by a physician specialized in radiology and brain imaging. All the subjects underwent a 6-minute echo-planar sequence in the resting state, using a multiband sequence with repetition time (TR)/echo time (TE)/precession angle = 720 ms/29 ms/44°; 48 slices; 500 volumes; acceleration factor 8; 82 × 82 matrixes; 268 mm field of view (FOV); and a 3 × 3 × 3 mm3 voxel size. In addition, an anatomical reference image was acquired with contrast T1 and a 3DMPRAGE sequence with TR/TE = 2200 ms/2.45 ms and a voxel size of 1 × 1 × 1. Three scans were excluded due to high in-scanner motion (defined as mean framewise displacement (FD) > 0.3 mm or maximum FD > 1.3 mm).

The anatomical data preprocessing of the T1-weighted (T1w) images was corrected for intensity nonuniformity (INU) with ‘N4BiasFieldCorrection’ distributed with ANTs 2.2.0. The T1w reference was then skull-stripped with a “Nipype” implementation of the ‘antsBrainExtraction.sh’ workflow (from ANTs) using OASIS30ANTs as the target template. Brain tissue segmentation into cerebrospinal fluid (CSF), white matter (WM), and gray matter (GM) was performed on the brain-extracted T1w image using ‘fast’ [FSL 5.0.9]. Volume-based spatial normalization to one standard space (MNI152NLin2009cAsym) was performed through nonlinear registration with ‘antsRegistration’ (ANTs 2.2.0) using brain versions of both the T1w reference and the T1w template. The following template was selected for spatial normalization: “ICBM 152 Nonlinear Asymmetrical template version 2009c” [MNI152NLin2009cAsym]. The blood oxygen level-dependent (BOLD) reference was then coregistered to the T1w reference using ‘flirt’ [FSL 5.0.9] with the boundary-based registration [@bbr] cost-function. Coregistration was configured with nine degrees of freedom to account for distortions remaining in the BOLD reference. Head-motion parameters with respect to the BOLD reference (transformation matrixes and six corresponding rotation and translation parameters) were estimated before any spatiotemporal filtering using ‘mcflirt’ [FSL 5.0.9]. The BOLD time series (including slice-timing correction when applied) were resampled onto their original, native space by applying the transforms to correct for head motion. These resampled BOLD time series are referred to as “preprocessed BOLD in original space” or just “preprocessed BOLD”. The BOLD time series were resampled into standard space, generating a “preprocessed BOLD run in [‘MNI152NLin2009cAsym’] space”. First, a reference volume and its skull-stripped version were generated using the custom methodology “fMRIPrep”. Several confounding time series were calculated based on the “preprocessed BOLD”: framewise displacement (FD), spatial deviation of successive difference images (DVARS) and three region-wise global signals. FD and DVARS were calculated for each functional run, both using their implementations in “Nipype”. The three global signals were extracted within the CSF, the WM, and the whole-brain masks. Additionally, a set of physiological regressors were extracted to allow for component-based noise correction [“CompCor”]. Principal components were estimated after high-pass filtering the “preprocessed BOLD” time series (using a discrete cosine filter with 128 s cut-off) for the two “CompCor” variants: temporal (tCompCor) and anatomical (aCompCor). tCompCor components were then calculated from the top 5% variable voxels within a mask covering the subcortical regions. This subcortical mask was obtained by heavily eroding the brain mask, which ensures that it did not include cortical GM regions. For aCompCor, the components were calculated within the intersection of the aforementioned mask and the union of the CSF and WM masks calculated in T1w space, after their projection to the native space (using the inverse BOLD-to-T1w transformation). The components were also separately calculated within the WM and CSF masks. For each CompCor decomposition, the “k” components with the largest singular values were retained, such that the retained components’ time series were sufficient to explain 50% of the variance across the nuisance mask (CSF, WM, combined, or temporal). The remaining components were dropped from consideration. Despiking was performed with AFNI’s 3DDESPIKE utility and the 36 parameters from the global confound regression.

## Atlas Selection

Each subject’s cortex was parcellated using the Power Functional Atlas and comprised 264 functional regions Regional BOLD time series were estimated by averaging time series over all voxels in each parcel Power (2011).

## Time Series Extraction and Graph Calculation

Time series of the fMRI data were decomposed on six levels using the maximum overlap discrete wavelet transform (MODWT), which allows signal decomposition at different resolution levels using the Daubechies least asymmetric wavelet filter (8). The decomposition was carried out with the R package *brainwaver* 1.6. Since the wavelet decomposition depends on the repetition time (720 ms), the maximum frequency obtained was 0.69 Hz. The relevant information was on decomposition level 4, corresponding to a 0.043–0.087 Hz frequency range (44).

From the signals obtained at decomposition level 4, the correlations (Pearson) between the time series of pairs of ROIs were calculated, and a correlation matrix per subject was built. The passing from a correlation matrix to an adjacent or binary matrix—to study connectivity patterns using the network theory—was achieved by choosing a threshold that allows establishing whether a particular connection was significant or not. The connection density used for this purpose in the present study was 5% (45). The procedure employed a connectivity conservation criterion by fixating the number of edges and chose a threshold that kept that number and varied for every matrix. Individual network measures were determined using the R *igraph* 1.0.1 package (46). Global efficiency was calculated to assess the functional organization of the networks. This measure quantifies the efficiency of information transmission between any of the nodes by multiple and parallel paths (47, 48). Global efficiency is the average of the efficiencies over all pairs of vertices and is denoted as follows:

(1) $$E=\frac{1}{n}\sum_{i\in N}E_{i}=\frac{1}{n}\sum_{i\in N}\frac{\sum_{j\in N,j\neq i}d_{ij}^{-1}}{n-1}$$

(49, 50) the distance *d_ij_* between any two vertices *i* and *j* in a graph is the number of edges in shortest path between *i* and *j*. An analog measure of global efficiency was used for the networks of interest (default mode network, salience network, and fronto-parietal network) where instead of *N* a subset of nodes *N_s_* was used, where these nodes belong only to the network of interest, and *n* was replaced by *n_s_*, which is the number of nodes in the network of interest. Making these substitutions in equation (1), we define the resulting *E*, which we call *E_s_*, to be the network efficiency, which only refers to information exchange between these subnetworks and not between these subnetworks and the whole brain.

Data were processed with the *IBM SPSS* 24.0 package for Windows. Descriptive statistics were used for demographically characterizing the sample and for describing the characteristics of the use of addictive substances. Normality and homoscedasticity were verified. The parametric Student’s t-test for independent samples was used for comparing the groups regarding the functional connectivity efficiency. The comparative analysis of cognitive functioning was achieved through the nonparametric Kruskal–Wallis test for independent test groups, owing to the noncompliance the assumption of normality for these variables and False Discovery Rate (FDR) correction for multiple comparisons to obtain adjusted *p*-values less than 0.05.

Once the descriptive analysis was performed and the intergroup differences on cognitive measures and on the connectivity of the three networks were determined, the second step was a mediation analysis to estimate the direct effects of the use of inhalants on the cognitive functions, as well as the mediation effects of the three functional networks between use and cognition. We regressed out the effects of nuisance covariates (sex, age, and other substance use) on dependent (Y) and mediating (M) variables. The residuals were then used in our mediation analysis. The significance of the indirect effect was evaluated using bootstrapped confidence intervals within the R package *lavaan*.

## Results

### Comparison Between Inhalant-Consuming and Nonconsuming Adolescents on Cognitive Performance

Analysis of the difference in means was performed using the Kruskal-Wallis test for independent samples since the condition of normality was not fulfilled. **Table 2** presents the means and standard deviation values for both groups. The cognitive performance of the IC group with respect to the HC group was assessed using these data while considering that due to the normalization of the scores, higher scores mean better executive performance.

**Table 2:** Executive Functioning. Descriptive statistics and differences between groups (Kruskal–Wallis test for independent samples).

Test	HC Mean (SD)	IC Mean (SD)
Working Memory Index	102.4 (13.2)	82.40 (8.2)*
Wisconsin Card	12.2 (2.9)	7.1 (3.1)*
Stroop	8.4 (4.9)	6.9 (4.3)*
Tower of Hanoi	9.8 (4.1)	7.2 (4.4)*

Significant differences between groups (*p* < 0.01) are indicated with *.

The performance of inhalant consumers was significantly lower than that of the controls in all cognitive tests. The IC presented a lower level of success on the WCST; that is, they generated fewer classification criteria. On the Stroop test, the consumers made more mistakes when they were asked to state the color of the word instead of reading it. They also needed more steps in the TOH test to solve the exercise, thus evidencing less efficiency in action planning and sequencing. Finally, the IC showed a lower index of verbal working memory, denoting a lower capacity to mentally maintain and manipulate verbal information.

### Comparison of the Global Efficiency and Network Efficiency: Default Mode Network, Salience Network, and Fronto-Parietal Network

**Figure 1:**
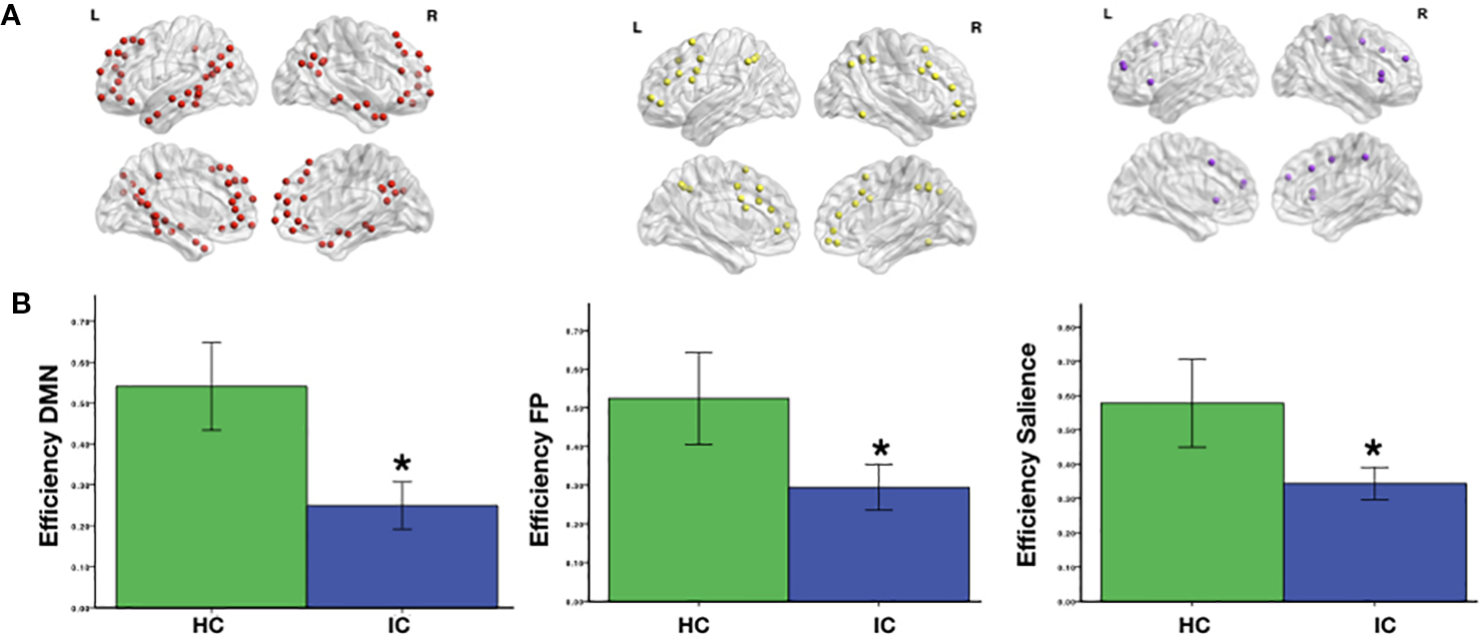
**(A)** Brain regions comprising the default mode (DMN), salience (SN), and fronto-parietal (FPN) networks according to the spatial coordinates from Power’s Functional Atlas (2011). **(B)** Significant differences (Student’s t-test) between the two groups (*_PFDR_ < 0.05) are indicated.

The network efficiency index showed significant levels of difference in means *p* < 0.05*_FDR_* for all three networks between the inhalant and nonconsumer adolescents. The DMN, however, showed the greatest difference between the two groups. The network efficiency scores, as shown in **Figure 1**, were significantly lower in inhalant-consuming adolescents than nonconsuming individuals. The global efficiency in the HC group was significantly greater than for the IC group (HC: 0.44 ± 0.21; IC: 0.26 ± 0.03 *p* = 0.0001).

### Mediation of the Relationship Between Inhalant Use and Cognitive Functioning by Network Efficiency

**Figure 2:**
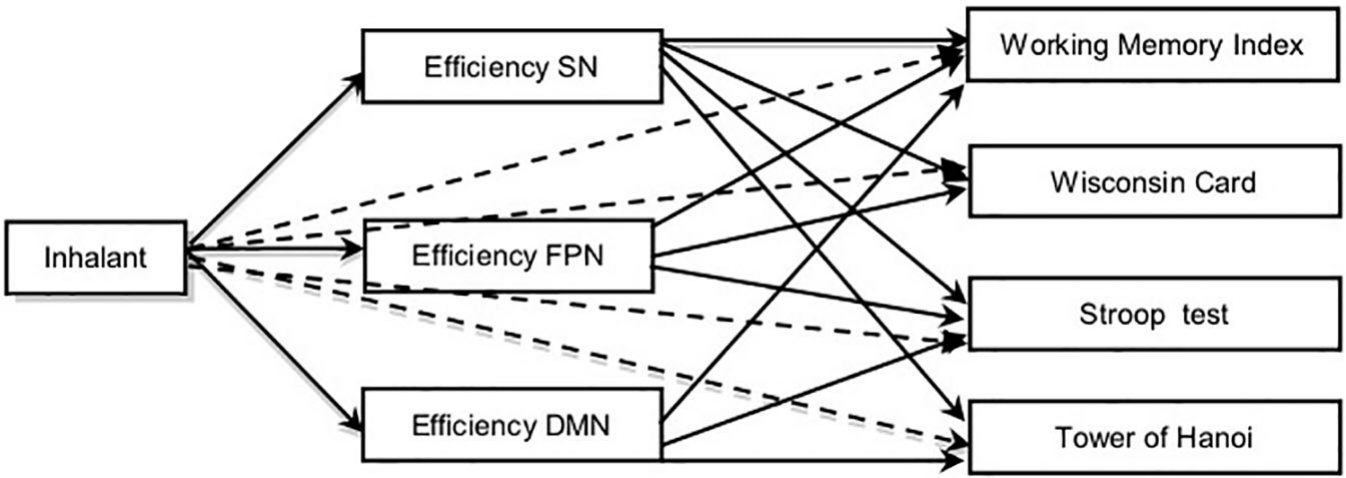
Graphic representation of a model on the effect of inhalants on cognitive functions. Discontinuous lines: direct effects of inhalant use.

**Figure 3:**
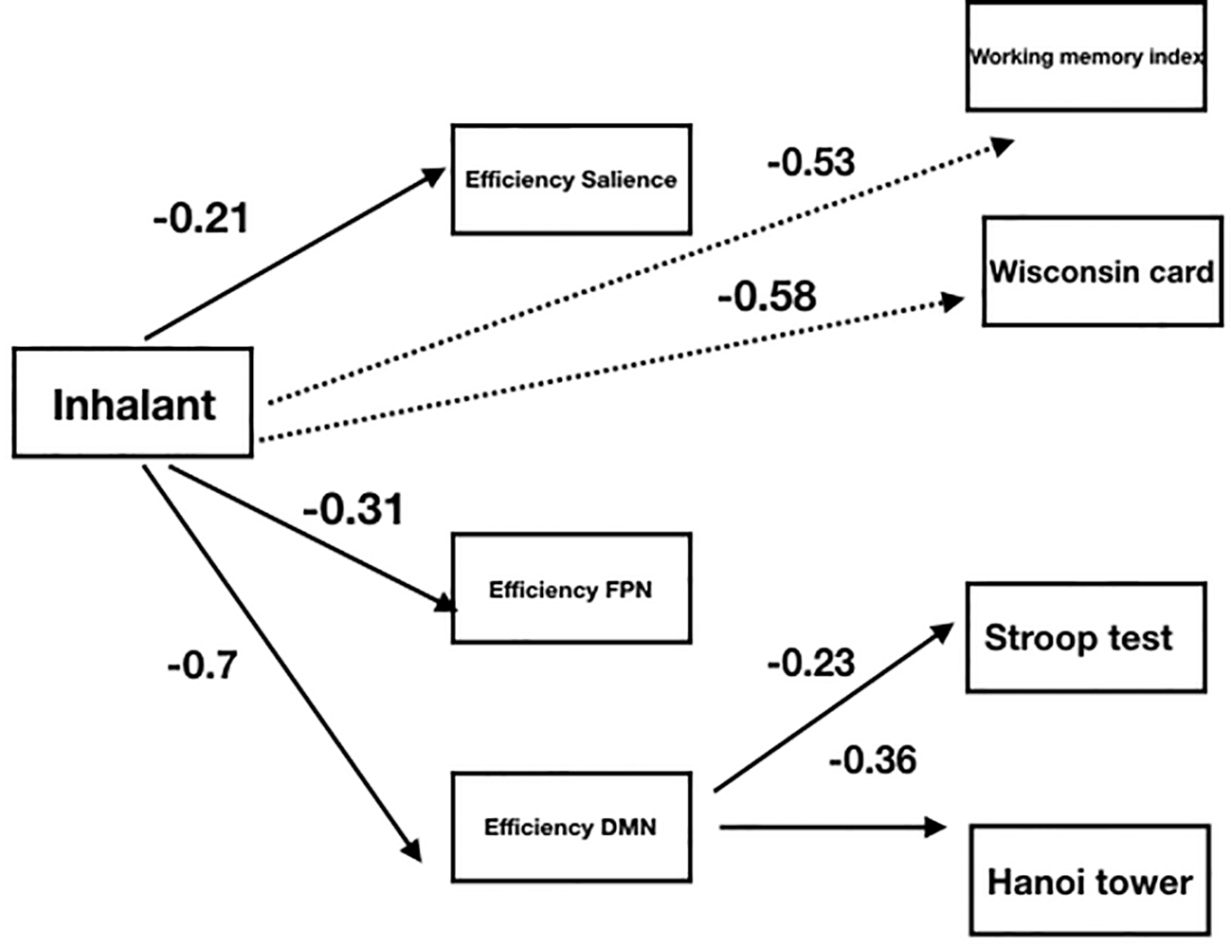
Graphic representation of an adjusted model using standardized coefficients(*p* < 0.05) and explained variance (*R*^2^) on the effect of inhalants on cognitive functions. Discontinuous lines: direct effects of inhalant use.

A path analysis was applied to explore whether the lower cognitive efficiency observed in IC subjects compared to noninhalant consumers may be related to differences in their functional brain communication. For that purpose, *lavaan* package was used. The variables of the model and their relationship were initially set in accordance with the working hypothesis (see **Figure 2**). The feasibility of using such a model was confirmed by the degrees of freedom ((Df > 0) = 10) involved in the comparisons, and the parameters were determined using the method of maximum likelihood. The model was adjusted on the basis of the modification indexes by eliminating the connections with nonsignificant coefficients (*p* > 0.05), and an adequate adjustment was achieved (X2 = 26.7, *p* = 0.11; CFI = 0.96; RMSEA = 0.05). The final adjusted model (**Figure 3**) differed from the initial one with the elimination of the assumption of a relationship between inhalant usage and the Stroop and TOH tests, as well as the elimination of the direct connections between the efficiency of the FPN and SN and the cognitive functions.

By considering the sign and magnitude of the estimated standardized parameters, the results showed that the use of inhalants had a negative effect on the efficiency of the functional connectivity in all three networks. Thus, the presence of inhalant use allowed for the prediction of a lower efficiency in communication between the brain regions that comprise these three networks. The strongest negative effect was observed towards the DMN, where the use of inhalants explained 30% of the variance.

Inhalant usage also has a direct negative effect on the working memory index and on the performance in the card sorting test, with coefficients of −0.53 and −0.58, respectively. The use of inhalants allowed the prediction of a lower capacity in the verbal working memory task and a lower performance in the card sorting test, which was used to assess the capacity of mental flexibility of an individual. No direct effects were observed between inhalant abuse and the Stroop and TOH tests.

When analyzing the role of connectivity efficiency as a mediator in the effects of inhalant use on cognitive performance, in opposition to the initial hypothesis, the analyzed data showed that the differences in cognitive performance were not mediated by how well connected the SN and FPN were. In contrast, the efficiency of the DMN did show a significant mediating effect, as this indirect path was the only explanation of the differences in performance in the Stroop and TOH tests between inhalant consumers and nonconsumers.

## Discussion

The present study compared complex cognitive functions in inhalant-consuming and nonconsuming adolescents. Both groups of individuals were also compared regarding the capacity of information exchange between nodes or regions of three functional networks DMN, SN, and FPN using the network efficiency and global efficiency obtained from the graph theoretical analysis. Finally, we explored whether network communication efficiency acted as a mediator of the effect of inhalant use on cognitive performance. The main findings were (1) a lower degree of working memory, mental flexibility, inhibitory control, and sequential planning in the IC group, (2) a lower functional connectivity efficiency for all three networks in the IC group, and (3) the occurrence of a mediator effect of the DMN between consumption and performance on the neuropsychological tests that explored inhibitory control and sequential planning functions.

For three networks, The IC group showed a lower network efficiency index when compared to the control group, thus evidencing a different functional organization characterized by a diminished capacity of information propagation and integration across the these networks. The executive function regulatory processes that monitor goal-directed cognitive operations are crucial for development to adulthood. The neural mechanisms underlying the normative maturation of executive functioning are related with segregation of networks modules and increase within-module connectivity mainly on DMN and FPN and further reciprocal activation FPN and DMN deactivation as well the flexible transition between this networks (28–30). Our study focused on four executive functions: verbal working memory, mental flexibility, inhibitory control and sequential planning. We found a significantly lower performance on tasks that measure each of these functions in inhalant-consuming adolescents than the controls. The cross-sectional design of the research is a limitation for analyzing whether the differences in cognitive functioning in the IC predated the use of drugs.

Recent longitudinal investigations (51, 52) have identified that weak working memory and poor impulse control can act as predictors of the progressive use of drugs in adolescents. Lower working memory capacity has been correlated with increased delay discounting and therefore has an effect on reward evaluation (53–55). Future longitudinal studies, which incorporate the assessment of different executive functions, might identify other cognitive predictors of addiction-related behaviors. Our data confirm the presence of alterations in cognitive functions that are fundamental for choosing and maintaining actions in everyday life (56, 57) in adolescents with inhalant use disorder and the need to include rehabilitation or cognitive stimulation among early intervention strategies.

Our results show that the DMN is the most affected network by inhalant abuse during adolescence. The mediation analyses suggested that the relationship between inhalant abuse and inhibitory control and sequential planning was partly mediated by DMN efficiency. Although this network has been mainly related to self-referential functions, numerous studies (58–60) have identified its indirect yet important contribution in the response to external cognitive demands. The high demands of executive functions require the availability of resources, which is achieved by diminishing the activation of the DMN. A deficit in the efficiency of the DMN in adolescents with inhalant use disorder might affect the induced deactivation of this network and hence compromise their executive functions (28). In favor of this hypothesis, there are recent data regarding the contribution of the DMN to the executive function deficit identified in patients with Alzheimer’s disease (59) and attention-deficit/hyperactivity disorder (61).

We must point out that the neuropsychological exploration performed here was focused on only four measures of executive functioning based on previous reports of these measures being affected in inhalant users (14, 39). It is possible that the detected decreased efficiency in the DMN, FPN, and SN may be responsible for other cognitive alterations and mental health problems also described among inhalant-abusing adolescents (62), which were not assessed in our research subjects.

According to the specific cognitive domains that have been linked to each network—the default network in self-referential functions, including autobiographical memory, the SN in identifying the most subjectively relevant stimuli, and the FPN in higher-order cognitive and attention control (63)—we expected to find a mediating effect of the FPN between inhalant abuse and poor performance in inhibitory control and sequential planning tasks. The absence of this effect may be related to the partitioning of the FPN into branches and the specific contribution of each sub-network in cognitive domains. (64) identified two main branches within the FPN: a dorsal spatial/motor network, which connects regions of the superior parietal lobe and the superior frontal lobe and a ventral non-spatial/motor network, which connects the inferior parietal lobe with the inferior and middle frontal gyri. When the FPN is analyzed based on its different components, we think that the behavior in these tests might be mediated by organizational characteristics of each branch instead of parameters of the overall FPN connectivity.

On the other hand, some authors have described the FPN as a flexible cognitive control center, with the ability to adapt its composition by recruiting diverse regions or networks, depending on the demands of the present task (65). We should also consider that each of the applied tests involves, in addition to the regions associated with the executive functions, other areas underlying different cognitive domains: the Hanoi Tower test involves regions associated with motor and visual/spatial abilities, while the Stroop tests involves regions that are associated with perception and verbal abilities. The nature of the executive demand determines the inclusion of the nodes in the FPN; therefore, the topological measurements may vary as a function of the accommodation of this functional structure. Furthermore, deficits associated with the inherent flexibility of the functional network for adapting its constitution as a response to the particular demands of each of the cognitive tests may be responsible for the differences in the executive behavior observed in the inhalant-consuming adolescents.

Finally, the IC group showed a lower global efficiency, similar results have been recently reported for alcohol-dependent patients. Sjoerds et al. (66) reported that resting brain functions were less efficient with longer alcohol dependence duration, while Wang et al. (67) found that brain networks of adult patients with alcohol use disorder showed decreased global efficiency compared to those of controls. Our results are consistent with previous research and suggest a diminishing in the efficiency of functional connectivity in individuals with SUDs. According to our research, this reduced global network efficiency might be present from an early age. Further recently study show that global efficiency mediated the improvement of executive functions with age in structural networks (29), however we do not have a global score of executive functions.

## Conclusions

This study explored novel neurocognitive aspects of addictive substance abuse, specifically inhalant use, during adolescence. Our findings show alterations in the topological organization of three functional brain networks of great importance for cognition: the DMN, the SN, and the FPN. Overall, we detected important variations in information transmission and integration between the brain regions that comprise these networks.

Important alterations in a number of executive functions were detected among the inhalant-consuming group, as well as the association between these alterations and the connectivity of the DMN. Taken together, our results demonstrate the usefulness of the analysis of functional brain networks in the resting state for improving the understanding of the changes in neural functioning underlying inhalant use disorder and suggest the need for its wider application in the field of SUDs. If these findings are validated in much larger samples, they could lead to the development of intervention strategies that consider the functional and structural brain plasticity present in adolescence, thus enabling the restoration of the functional architecture of the affected brain networks.

## Limitations

Limitations of this research include the following:

1. Throughout the paper we focus in the default, fronto-parietal, and salience networks. However, it is known that brain topology of other systems relating to cognitive control and attention, such as the Cingulo-Opercular, Dorsal Attention, and Ventral Attention networks are directly involved in executive functions. It would be desirable to explore these correlations in a future study.
2. Its transversal design prevents establishing causality between inhalant use and the efficiency of functional connectivity, as well as between inhalant use and the state of executive brain functions.
3. The modest size of the sample limits the analysis of the results and the reach of its interpretation.
4. Some variables, such as the socioeconomic status of the subjects, the quality of their educational institutions, the presence of family stress and other variables related to their family history having a potential impact on the brain and cognitive development, were not considered.
5. Substance use history was established from an interview, where the subjects may have minimized their use of inhalants or other drugs.
6. Most of the subjects were on initial remission under pharmacological treatment, and both conditions may have affected cognitive performance.
7. Many inhalant users also consumed other substances, although to a lesser extent. The results must accordingly be interpreted while taking into account the occurrence of polydrug use, which is characteristic of this population.

Finally, our results should be considered within the limits of the adolescence period, which is an important stage for brain development. The study of the difference in the topological organization of brain networks and its impact on cognitive functioning in adult inhalant users may lead to different results and constitute a future line of research.

## Data Availability Statement

The datasets analyzed in this article are not publicly available as the participants did not give their informed consent for the public availability of their data. Requests to access the datasets should be directed to NG-G, nagonzalez@himfg.edu.mx.

## Ethics Statement

The studies involving human participants were reviewed and approved by the Ethics Committee of the Children’s Hospital of Mexico “Federico Gomez”. Written informed consent to participate in this study was provided by the participants’ legal guardian/next of kin.

## Author Contributions

NG-G, DH-A, and CT-R conceived the study and participated in the data collection. NG-G, DH-A, LP, and RV-S analyzed the data and carried out the statistical analysis. DH-A, NG-G, and MP wrote the manuscript. All authors have approved the final manuscript.

## Funding

This study was carried out thanks to the support and financing of the following:

- National Council of Science and Technology CONACYT. No. support 412003.
- National Council of Science and Technology CONACYT-FOSSIS 2012-01-182160.
- Children’s Hospital of Mexico “Federico Gómez”. HIM Protocol 2016-016 SSA-1251.
- Institute of Cellular Physiology of the UNAM. Projects (DGAPA) PAPIIT IN 205217.
- UNAM-PAPIIT TA100620.

## Conflict of Interest

The authors declare that the research was conducted in the absence of any commercial or financial relationships that could be construed as a potential conflict of interest.
